# Alimentary Tract Bacteria Isolated and Identified with API-20E and Molecular Cloning Techniques from Australian Tropical Fruit Flies, *Bactrocera cacuminata* and *B. tryoni*

**DOI:** 10.1673/031.010.13101

**Published:** 2010-08-12

**Authors:** N. Thaochan, R. A. I. Drew, J. M. Hughes, S. Vijaysegaran, A. Chinajariyawong

**Affiliations:** ^1^ Institute of Agricultural Technology, Walailak University, Nakhon Si Thammarat, 80161, Thailand; ^2^ Center for Agricultural Biotechnology (AG-BIO/PERDO-CHE), Thailand; ^3^ International Centre for the Management of Pest Fruit Flies, Griffith School of Environment, Griffith University, Nathan campus, Nathan, Queensland, 4111, Australia; ^4^ Griffith School of Environment, Griffith University, Nathan campus, Nathan, Queensland, 4111, Australia

**Keywords:** *Bactrocera cacuminata*, *Bactrocera tryoni*, PCR, 16S rRNA gene, *Enterobacteriaceae*, lactic acid bacteria

## Abstract

Bacteria were isolated from the crop and midgut of field collected *Bactrocera cacuminata* (Hering) and *Bactrocera tryoni* (Froggatt) (Diptera: Tephritidae). Two methods were used, firstly isolation onto two types of bacteriological culture media (PYEA and TSA) and identification using the API-20E diagnostic kit, and secondly, analysis of samples using the 16S rRNA gene molecular diagnostic method. Using the API-20E method, 10 genera and 17 species of bacteria in the family Enterobacteriaceae were identified from cultures growing on the nutrient agar. The dominant species in both the crop and midgut were *Citrobacter freundii, Enterobacter cloacae* and *Klebsiella oxytoca. Providencia rettgeri, Klebsiella pneumoniae* ssp *ozaenae* and *Serratia marcescens* were isolated from *B. tryoni* only. Using the molecular cloning technique that is based on 16S rRNA gene sequences, five bacteria classes were dignosed — Alpha-, Beta-, Gamma- and Delta- Proteobacteria and Firmicutes — including five families, Leuconostocaceae, Enterococcaceae, Acetobacteriaceae, Comamonadaceae and Enterobacteriaceae. The bacteria affiliated with Firmicutes were found mainly in the crop while the Gammaproteobacteria, especially the family Enterobacteriaceae, was dominant in the midgut. This paper presents results from the first known application of molecular cloning techniques to study bacteria within tephritid species and the first record of Firmicutes bacteria in these flies.

## Introduction

The bacteria associated with tephritid fruit flies have been widely studied using traditional microbiological methods where the gut microflora is cultured on specific nutrient agar media and subsequently identified by phenotypic characterization of isolates using the API-20E system (Drew et al. 1983; Kuzina et al. 2001; Marchini et al. 2002). The API-20E system is suitable for the identification of enteric bacteria and provides a convenient method to inoculate and read tests relevant to members of the family Enterobacteriaceae and associated organisms. Consequently, a large number of studies have been conducted on bacteria isolated from the alimentary tract of fruit flies using the API-20E system resulting in identification of bacteria mainly belonging to the family Enterobacteriaceae with the species most commonly identified as *Citrobacter freundii, Enterobacter agglomerans, Enterobacter cloacae, Providencia rettgeri* and *Klebsiella oxytoca* (Capuzzo et al. 2005; Drew et al. 1983; Drew and Lloyd 1987; Fitt and O'Brien 1985; Howard et al. 1985; Kuzina et al. 2001; Lloyd et al. 1986; Murphy et al. 1994).

However, besides the Enterobacteriaceae, little is known of other species of bacteria in other families that may also inhabit the alimentary tract of fruit flies. Molecular approaches for the detection and characterization of microbes in other insect species have revealed considerable bacterial diversity (Friedrich et al. 2001; Haynes et al. 2003; Schmitt-Wagner et al. 2003). In particular, nucleic acid sequence approaches of 16S rRNA genes are now revealing considerable new data on the microbial community of insects (Brauman et al. 2001). For example, considerable research using both culture-dependent and culture-independent techniques has been conducted on diagnosing the gut bacteria of Coleoptera (Delalibera et al. 2007; Vasanthakumar et al. 2006, 2008). Studies on the adult southern pine beetle, *Dendroctonus frontalis*, and the adult pine engraver, *Ips pini*, revealed that their bacterial gut communities have a relatively low species richness. In the adult emerald ash borer, a *Agrilus planipennis*, more diverse bacterial community was detected and, in all three cases, a higher diversity of bacteria was detected by the analysis of 16S rRNA gene sequences of gut isolates. To date, recent molecular diagnostic techniques have not been employed to identify the alimentary tract bacteria of fruit flies.

This paper presents results of research carried out on alimentary tract bacteria of two Australian fruit fly species, *Bactrocera cacuminata* (Hering) and *Bactrocera tryoni* (Froggatt) (Diptera: Tephritidae), identifying the bacteria using both the API-20E system and 16S rRNA gene molecular analyses, and based on these results, an analysis of bacteria species diversity and community similarity in these two species of fruit flies.

## Methods and Materials

### Fruit fly collecting and handling

Adult flies of *B. cacuminata* and *B. tryoni* were hand collected from fruiting host plants in Brisbane, Queensland, Australia during the months of February and March, 2007. Specimens of *B. cacuminata* were collected from wild tobacco, *Solanum mauritianum* Scopoli and *B. tryoni* from custard apple (*Annona reticulata* L.), guava (*Psidium guajava* L.) and loquat (*Eriobotrya japonica* (Thunberg) (Lindl.). Captured flies were held individually in clear plastic vials to prevent cross-contamination of bacteria between flies. The vials containing flies were plugged with cotton wool for ventilation and placed in a cool ice box in the field to immobilize them and to prevent flies from regurgitating their crop contents within the tubes.

### Dissection and isolation of bacteria from the alimentary tract of fruit flies

Five males and five females of each species of the field-collected fruit flies were killed immediately on return to the laboratory by freezing at -20° C for 3 min. Flies were then surface sterilized by immersing in 70% ethanol for 1 min, 0.5% sodium hypochlorite for 1 min and then washed twice in sterile distilled water (modified from Lloyd 1991). The surface-sterilized flies were individually dissected under sterile distilled water in a sterile glass cavity block. Before dissecting, the water in each glass cavity box was sampled and spread onto tryptone soya agar (TSA) (Oxoid) and peptone yeast extract agar (PYEA) (Oxoid, www.oxiod.com) and incubated at 35° C for 24–48 h to determine whether any contaminant bacteria were present. The fruit fly sample was discarded if contamination occurred on the media. Although PYEA and TSA are recognised as media that grow similar groups of microorganisms, it was decided to use both in this study to maximise the chance of isolating most of the bacteria species in the fly.

The crop and midgut of each fly were aseptically removed following the method described by Drew et al. (1983) and Lloyd (1991), and placed in a sterile 1.5 ml microcentrifuge tube and homogenized with a sterile inoculation loop. These contents were spread onto TSA and PYEA and incubated at 35° C for 24–48 h. All steps in the isolation procedure were performed in a laminar flow hood to avoid aerial contamination. To avoid cross-contamination between the different gut regions, the crop was removed first by pinching the narrow entry tube and lifting it out, and then removing the midgut. If either organ broke open before removal, that fly was discarded. A qualitative assessment of the numbers and types of colonies growing on each plate was made after 24 and 48 hours, and the predominant types were purified through repeated subculturing. The method of purification was as follows: At the end of the incubation period, each bacterial colony was aseptically removed by using an inoculation loop, spread onto TSA and PYEA and incubated aerobically at 35° C for 24–48 h. Each colony was isolated on the basis of morphological appearance and sub-cultured twice to ensure purity.

### Identification of bacteria isolates with API-20E

All bacteria isolates were initially Gram-stained for Gram-positive and Gram-negative identification and tested for oxidase/catalase activity. Gram negative and rod shaped bacteria were chosen for identification with the API-20E system (bioMerieux sa 62980, www.biomerieux.com). Analytical Profile Indexes from the API-20E system were used for diagnosing species within the family Enterobacteriaceae only. The ID profiles were rated from excellent to good, based on the API codes.

### Molecular cloning study of the 16S rRNA gene of gut bacteria community in the fruit fly alimentary canal.

Steps in obtaining the crops and midguts of the flies for the molecular cloning study were the same as those described above.

Three flies of each sex were dissected under sterile distilled water. The crop and midgut were removed separately and placed into sterile 1.5 ml centrifuge tubes. DNA was extracted using a modification of the CTAB/phenol-chloroform DNA extraction protocol (Doyle and Doyle 1987).

Extracted DNA from the crop and midgut were combined and the fragments were amplified in a polymerase chain reaction (PCR) using the universal primers for bacteria, forward primer Y1 (5′-TGGCTCAGAACGAACGCTGGCGGC-3′) (Sigma, www.sigmaaldrich.com) and reverse primer Y2 (5′-CCCACTGCTGCCTCCCGTAGGAG T-3′) (Sigma) (Young, Downer & Eardly, 1991). The reactions were carried out in a 100 µl volume containing 2 µl of template DNA solution, 2 µM of the primer, 200 µM of deoxynucleosidetriphosphate (Astral Scientific, www.astralscientific.com; Bioline, www.bioline.com) and 2 U of *Tag* DNA polymerase (Astral scientific, Bioline). The amplifications were performed using the following protocol: initial denaturation at 94° C for 5 min; 30 cycles of 45 s at 94° C, 40 s at 62° C and 2 min at 72° C, and final extension at 72° C for 10 min. After the reaction, 5 µl aliquots of PCR products were examined by electrophoresis in 1% agarose gel. The PCR products were extracted with QIA quick gel extraction kit (Qiagen, www.qiagen) for ligation.

The ligations were performed in 10 µl containing 1 µl pDrive cloning vector (50 ng/µl), 2.5 µl mix DNA, 1.5 µl distilled water, and 5 µl 2x ligation master mix (Qiagen) and introduced into *Escherichia coli* (strain JM109, Promega, www.promega.com) by transformation. The recombinants were selected and verified at the correct insert size by vector-targeted PCR with primer M13 F (5′-GTAAAACGACGGCCAGT-3′) (Sigma) and M13 R (5′CAGGAAACAGCTATGAC-3′) (Sigma) by the following PCR protocol: an initial denaturation at 94° C for 4 min; 35 cycles of 30 sec at 94° C, 40 sec at 53° C and 60 sec at 72° C. Finally, samples were subjected to 72° C for 4 min and then held for an indefinite period at 4° C. From each clone library, 30 clones were randomly selected and sequenced.

### Classification of sequences and phylogenetic analyses

The bacteria were diagnosed by uploading the sequences obtained from the 16S rRNA analyses onto Ribosonal Database Project II (RDP-II database) (Wang et al. 2007), and classified using the classifier tool. Further, a phylogenetic tree was constructed incorporating the bacteria diagnosed from the fruit flies and related type strains on the RDP database. This was achieved by using the Molecular Evolutionary Genetics Analysis (MEGA) version 4 (Kumar et al. 2008) to align the sequences and construct a neighbour — joining bootstrap tree utilizing Kumar's two-parameter model (Nei and Kumar 2000).

### Morista index and rarefaction analyses

Morista index values of isolates diagnosed by both the API-20E and molecular cloning methods were calculated after the method of Krebs (1998). In addition, rarefaction curves were drawn for isolates with a 95% or more similarity, based on the 16S rRNA sequence data. For this, analytical rarefaction software was used (version 1.2; S.M. Holland, University of Georgia, Athens, Ga; http://www.uga.edu/strata/software.html) (Schmitt-Wagner et al. 2003).

## Results

### Overview of bacteria colonies isolated by dissection and culturing of alimentary parts

Of the two bacterial culture media used, more bacteria colonies grew on TSA than PYEA, while on both media more bacteria species were recovered from the fly midgut than the crop. A total of 125 bacteria colonies were isolated (Table 1), 49 from *B. cacuminata* (29 of these on TSA and 20 on PYEA) and 76 from *B. tryoni* (44 isolates on TSA and 32 on PYEA). The physical characteristics of the colonies were similar when growing on both culture media, most being cream with a few red and yellow colonies. No fungi and yeasts were recovered. Gram negative and rod shaped bacteria were the major microorganisms found in both species of fruit flies.

**Table 1.**
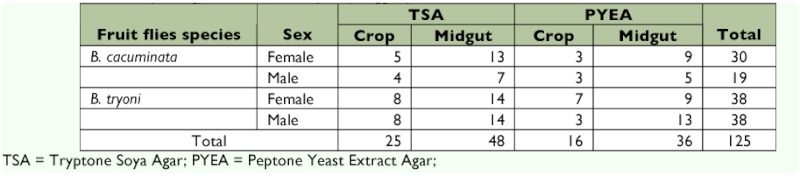
Summary of the number of bacteria colonies isolated from the crop and midguts of field collected adults of *Bactrocera cacuminata* (Hering) and *Bactrocera tryoni* (Froggatt) on two different culture media, TSA and PYEA.

**Table 2.**
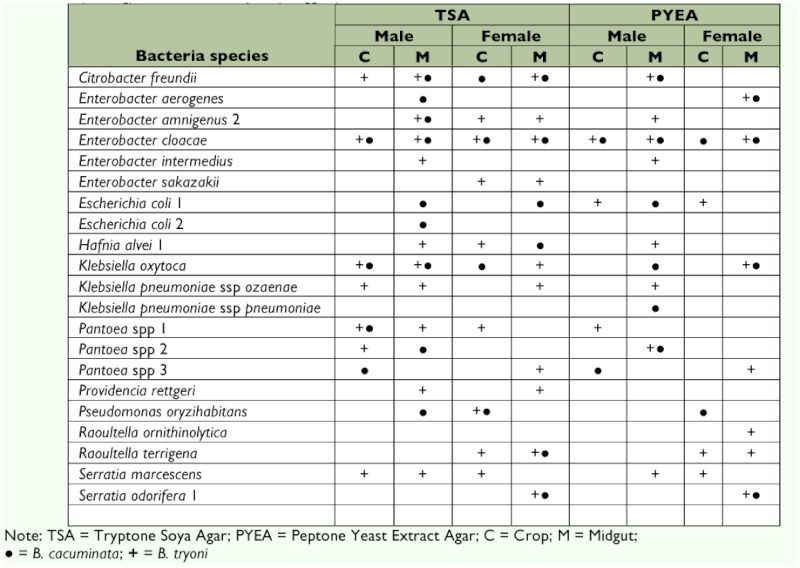
Genera and species of bacteria in the family *Enterobacteriaceae*, isolated from field collected adults of *Bactrocera cacuminata* (Hering) and *Bactrocera tryoni* (Froggatt) and identified with API-20 E.

### Bacteria identification with API-20E

Identification using the API-20E system diagnosed one family, Enterobacteriaceae, with 10 genera and 17 known species of bacteria. The genera were *Citrobacter, Enterobacter, Escherichia, Hafnia, Klebsiella, Pantoea, Providencia, Pseudomonas, Raoultella* and *Serratia* (Table 2).

*Citrobacter freundii, Enterobacter cloacae* and *Klebsiella oxytoca* were the most commonly occurring bacteria isolated from both *B. cacuminata* and *B. tryoni*, with *Escherichia coli, Pantoea* spp. and *Klebsiella pneumoniae* ssp *ozaenae* being less frequent.

*Enterobacter cloacae* grew on TSA and PYEA from the crop and midgut of *B. cacuminata* but was not detected on PYEA from the crop of female *B. tryoni. Enterobacter intermedius, Enterobacter sakazakii, K. pneumoniae* ssp *ozaenae, Providencia rettgeri*, *Raoultella ornithinolytica* and *Serratia marcescens* were isolated only from *B. tryoni. Providencia rettgeri* was found in the midgut of both male and female *B. tryoni* on TSA only. *Klebsiella pneumoniae* ssp *ozaenae* and *S. marcescens* were frequently observed in crop and midgut isolates of either male or female *B. tryoni* on both culture media (Table 2).

### Identification of bacteria by molecular cloning of the 16S rRNA gene

The molecular cloning of the 16S rRNA gene, from bacteria present in the crops and midguts of both fruit fly species, identified the presence of some bacteria species that were not detected through the culturing of the crop and midgut contents on TSA and PYEA and subsequently identified using the API-20E method (Table 3).

Four clone libraries of 16S rRNA were assembled, each representing either the crop or midgut of the two fly species (Table 3). There were 35 clones affiliated with Firmicutes, 40 affiliated with Gammaproteobacteria, 5 affiliated with Alphaproteobacteria and one each of Betaproteobacteria and Deltaproteobacteria (Table 3). The percentage incidence of each clone in each bacterial family is presented in Table 3.

**Figure 1.**
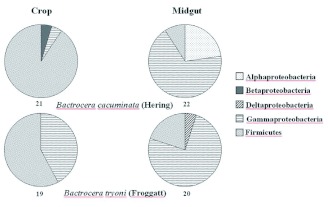
Percent incidence of Alpha, Beta, Delta and Gamma Proteobacteria and Firmicutes in the crop and midgut of *Bactrocera cacuminata* (Hering) and *Bacterocera tryoni* (Froggatt). The total number of clones in each clone library is given under each pie chart. High quality figures are available online.

The crop of *B. cacuminata* had 21 clones which belonged to three bacterial classes, Firmicutes, Gammaproteobacteria and Betaproteobacteria and three bacterial families, Leuconostocaceae, Enterobacteriaceae and Comamonadaceae, while the midgut had 22 clones from three classes, Firmicutes, Alphaproteobacteria and Gammaproteobacteria, and three families, Leuconostocaceae, Acetobacteriaceae and Enterobacteriaceae. The crop of *B. tryoni* had 19 clones from two classes, Firmicutes and Gammaproteobacteria, and three families, Enterococcaceae, Leuconostocaceae and Enterobacteriaceae, while the midgut had 20 clones from three classes, Firmicutes, Gammaproteobacteria and Deltaproteobacteria, and two families, Enterococcaceae and Enterobacteriaceae (Table 3).

**Table 3.**
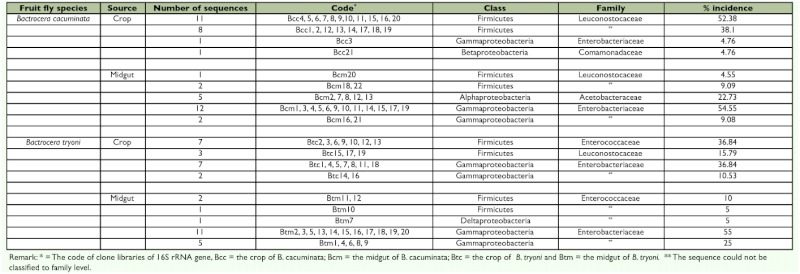
Classes and families of bacteria diagnosed by analysing the sequences of the 16S rRNA gene, obtained from the crops and midguts of field collected adults of *Bactrocera cacuminata* (Hering) and *Bacterocera tryoni*

The relative clone frequencies are illustrated in Figure 1. In the crop of both fruit fly species, Firmicutes was the dominant bacterial class. The crop of *B. cacuminata* contained more bacterial classes than the crop of *B. tryoni* but Betaproteobacteria was found only in the crop of *B. cacuminata*. In the midgut of both fruit fly species, Gammaproteobacteria was the predominant bacterial class. Deltaproteobacteria was found only in the midgut of *B. tryoni*.

### Lactic acid bacteria in the alimentary tract of adult fruit flies

Clones obtained from the crop of each species of fruit fly were mainly gram-positive bacteria and these were less frequently found in the midgut. Most clones belonged to the division Firmicutes, class Bacilli, order Lactobacillales, families Enterococcaceae and Leuconostocaceae. These bacteria families, called lactic acid bacteria (*Holzapfel and Wood 1998*), were particularly common in the crops of *B. cacuminata* (90.48%) and *B. tryoni* (52.63%) (Table 3).

### Phylogenetic analysis

**Figure 2.**
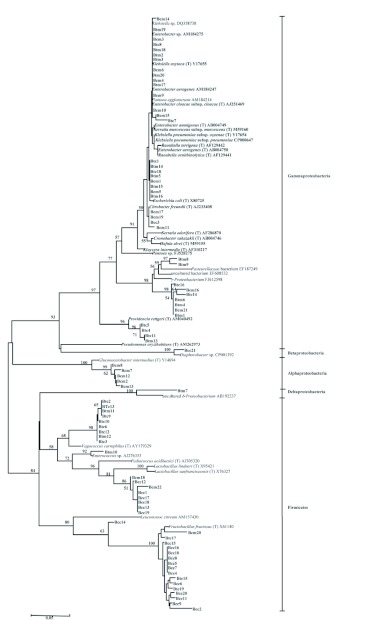
Phylogenetic tree based on 16S rRNA gene sequences from bacteria obtained from crops and midguts of two fruit flies species, *Bactrocera cacuminata* (Hering) and *Bactrocera tryoni* (Froggatt). Code: Bcc, the crop of *B. cacuminata*, Bcm, the midgut of *B. cacuminata*, Btc, the crop of *B. tryoni* and Bcm, the midgut of *B. tryoni*. The species identified by the API-20E strips and the RDP-II database are in bold type. Other codes refer to the species identified through other clone libraries. The scale bar indicates evolutionary distance (5 substitutions per 100 nucleotides). High quality figures are available online.

The phylogenetic tree based on the 16S rRNA sequences of bacteria species from both *B. cacuminata* and *B. tryoni* is presented in Figure 2. Sequences aggregated into five clusters in conformity with the bacterial classes Gammaproteobacteria, Alphaproteobacteria, Betaproteobacteria, Deltaproteobacteria and Firmicutes. In Gammaproteobacteria, many clones were affiliated with known species such as *Enterobacter* sp., *Enterobacter aerogenes, Klebsiella oxytoca, Escherichia coli, Citrobacter freundii, Providencia rettgeri* and unclassified Gammaproteobacterium. In Alphaproteobacteria, a few clones were affiliated with *Gluconacetobacter intermedius*. Furthermore, in Betaproteobacteria and Deltaproteobacteria clusters, one clone of each was affiliated with *Diaphorobacter* sp. and an uncultured Deltaproteobacterium, respectively. Finally, in the last cluster Firmicutes, many clones were affiliated with *Vagococcus carniphilus, Lactobacillus* sp. and *Fructobacillus fructosus*, while one clone was affiliated with *Enterococcus* sp.

### Morisita index and rarefaction analyses

Bacteria species isolated and identified with the API-20E method showed relatively high Morisita index values indicating that most species present in the crops and midguts of both sexes of each fly species were similar. Five of the six indices were more than 0.600 and this result would be expected as the API-20E diagnostic method primarily identifies species of the family Enterobacteriaceae (Table 4).

The Morisita index, based on the 16S rRNA sequence data, was categorized into three levels each with a sequence similarity ≥80%, ≥90% and ≥95% and which corresponded with the phylum, class and genus levels (Table 5). At the phylum level, the community similarity values (crop vs crop and midgut vs midgut) between the different fruit fly species had high values of 0.807 and 1.000 respectively (Table 5A). However, different parts of the alimentary tract within the same fruit fly species showed low Morisita index values, 0.262 and 0.363 (Table 5A). Comparison at class level for the same alimentary tract portion between the different fly species showed Morisita indices similar to the phylum level with 0.769 (crop vs crop) and 1.000 (midgut vs midgut) (Table 5B).

However, when comparing the different alimentary tract parts in the same fly species, the value was low in *B. cacuminata* (0.165) and high in *B. tryoni* (0.874) (Table 5B). Furthermore, when the index was assessed at the genus level, the values for the different alimentary tract portions in the same fly species were 0.407 and 0.765, while for the same alimentary tract portion between the different fruit fly species, the value was zero (Table 5C).

**Figure 3.**
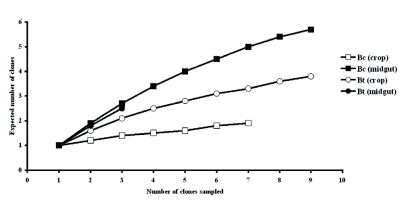
Rarefaction curves based on 16S rRNA gene clones recovered from the crops and midguts of *Bactrocera cacuminata* (Hering) (Bc) and *Bactrocera tryoni* (Froggatt) (Bt). High quality figures are available online.

The rarefaction curves based on 95% similarity of taxonomic units (Figure 3), demonstrated steeper inclines for the microflora of the midgut than the crops of both fruit fly species. While all curves indicated a very high level of microbial diversity, the highest level was found in the midgut of *B. cacuminata*.

**Table 4.**
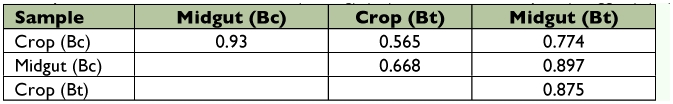
Comparison of Morisita index values of community similarity of bacteria identified with the API 20 E system, isolated from the alimentary tract of *Bactrocera cacuminata* (Hering) (Bc) and *Bacterocera tryoni* (Froggatt) (Bt).

**Table 5.**
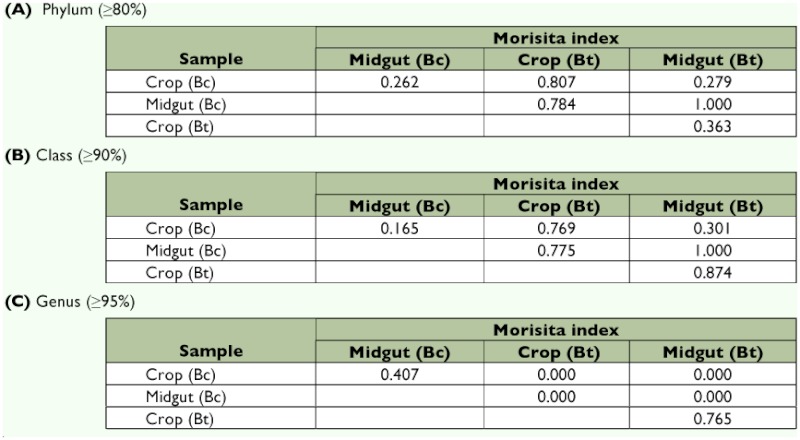
Morisita index values based on 16S rRNA gene sequences from the crops and midguts of *Bactrocera cacuminata* (Hering) (Bc) and *Bactrocera tryoni* (Froggatt) (Bt) at three taxonomic levels — Phylum, Class and Genus.

## Discussion

There are no reports in the literature on the molecular diagnosis of bacteria associated with fruit flies. All previous studies have been based on API-20E identifications (Drew & Lloyd 1989, 1991). The two culture media used in this study, TSA and PYEA, in general produced the same species of bacteria although TSA was slightly more productive in numbers of isolates. However, the molecular analyses of crop and midgut contents demonstrated that other bacteria species, which do not grow on TSA and PYEA media, were also present. A similar study of soil bacteria revealed that TSA medium did not facilitate the isolation of some less common bacteria groups but did produce the more common ones (Davis et al. 2005).

Using the physical dissection technique, 125 bacteria colonies were isolated and identified by the API-20E identification method. This resulted in the diagnosis of bacteria belonging to a single family, the Enterobacteriaceae. Many bacterial species, including *E. cloacae, C. freundii, K. oxytoca, K. pneumoniae* spp. and *Serratia* spp., in this family, are common microflora in the alimentary canal of insects (Moreira et al. 2005; Pai, Chen and Peng 2004) and also in different species of tephritid flies (Kuzina et al. 2001; Marchini et al. 2002). Of the 17 known bacteria species diagnosed in this study, 6 occurred in *B. tryoni* only and two in *B. cacuminata*, while the remaining 9 were common to both fruit fly species. Species of bacteria that were isolated from fruit flies and studied by Lloyd et al. (1986), namely *C. freundii, E. cloacae* and *K. oxytoca* were also found in *B. cacuminata* and *B. tryoni* in this study, while *P. rettgeri* was only isolated from *B. tryoni*. In general, more bacteria species resulted from culturing on TSA medium than on PYEA. In comparing the bacteria species present in the crop and midgut of *B. tryoni*, there was a high level of similarity in contrast to a low level in the same comparison within *B. cacuminata.* Lloyd et al. (1986) reported that some genera of bacteria such as *Serratia* are not part of the common flora of the alimentary tract of wild adults as they were only detected from the alimentary tract of mass cultured laboratory flies. In our study *Serratia* was found in both field collected *B. cacuminata* and *B. tryoni*.

The 16S rRNA sequence data of the whole crop and midgut of the two species of fruit flies resulted in 5 classes and 5 families of bacteria being diagnosed. Within *B. cacuminata* there were 3 classes and 3 families in both the crop and midgut while in *B. tryoni*, 2 classes and 3 families in the crop and 3 classes and 2 families in the midgut. Across both fruit fly species, the class Firmicutes (family Leuconostocaceae) contained the predominant clones in the crop while the class Gammaproteobacteria clones were predominant in the midgut, particularly within the family Enterobacteriaceae. Cox and Gilmore (2007) reported that the gut microbial flora in the ferment fly, *Drosophila melanogaster*, consisted of 37.3% bacteria species in the division Firmicutes, particularly the genus *Enterococcus*. Bacteria belonging to the classes of Proteobacteria comprised 61% of the microbial flora, with the common genera being *Acetobacter, Gluconobacter, Wolbachia, Enterobacter, Klebsiella, Pantoea, Citrobacter, Erwinia, Serratia, Morganella, Pesudomonas* and *Stenotrophomonas*.

The presence of species of Firmicutes and Proteobacteria in the crop and the midgut of *D. melanogaster* was related to the pH within the alimentary tract (Cox and Gilmore 2007). The crop of fruit fly species was found to be acidic with a pH 3–3.5 (Drew et al. 1983) whilst the midgut was alkaline with pH 5.7–7.4, although the midgut was reported to be strongly acidic, with a pH of 1.4–2.0 in *B. tryoni* (Fitt and O'Brien 1985). In the case of *B. tryoni*, Murphy et al. (1994) showed that the main site of bacteria colonization was the midgut lumen, inside the peritrophic membrane. In other studies on *Drosophila* species, similar pH levels to those reported by Drew et al. (1983) for *B. tryoni* were found, i.e., an acidic crop, a highly alkaline ventriculus and a neutral to acidic hindgut (Clark 1999). The pH is important for the selection and growth of certain species of bacteria. Most bacteria have an optimum pH for growth of pH 6–7, but an exception is the lactic acid bacteria that can grow in acidic conditions (Dillon and Dillon 2004). The presence of Firmicutes in the crops and Proteobacteria in the midguts, in our study, is consistent with these optimum pH levels.

The presence of lactic acid bacteria in insects has been reported in several species of wood and soil-feeding termites (Bauer et al. 2000; Miyata et al. 2007) and some Lepidopteran species in which they formed the majority of carbohydrate-utilizing bacteria in the hindgut (Shannon et al., 2001).

Lactic acid bacteria have been diagnosed in fruit flies of the genus *Bactrocera* for the first time in this study. Four genera were present, *Lactobacillus, Leuconostoc, Pediococcus* and *Vagococcus*. These genera are important members of Firmicutes and species within this group are usually found in decomposing plants where they produce lactic acid as the major metabolic end product of carbohydrate fermentation. For example, *Leuconostoc fructosum* (AF360737) has been recorded in ripe fig in nature (Antunes et al. 2002).

Based on the API-20E data, the community similarity measurement (Morisita index) of the isolated gut bacteria had a percentage identity of more than 50% for each fraction of gut content and each sex of the two fruit fly species. Because this analysis was based on the Enterobacteriaceae, the primary bacteria diagnosed through the API-20E system, this community similarity measurement would be expected. The Morisita index analysis of the bacteria from the crops and midguts of the flies confirmed that, at the bacteria species level, the fruit fly species were different, but similar at the bacteria family and order level. Based on the 16S rRNA gene sequence data, the midgut of the two fruit flies species had a Morisita index value of 1.000 at both the Phylum and Class levels, confirming that both species possessed similar higher taxonomic level gut bacteria communities. However, at the genus and species level they were considerably different, with *B. cacuminata* having a low level of similarity between the crop and midgut (Morisita index 0.407) and *B. tryoni* possessing a high level of similarity (Morisita index 0.765) between these organs. When comparing *B. cacuminata* and *B. tryoni*, crop vs crop and midgut vs midgut, the Morisita index was zero at the bacteria genus level, proving a high level of dissimilarity.

The *Bactrocera* species possessed two major classes of bacteria in their alimentary canals, Firmicutes and Proteobacteria, the same as those reported in *Drosophila* (Cox and Gilmore 2007). The Firmucutes were found mainly in the crop of the flies that possess a low pH (Drew et al. 1983). In some other insects, e.g. termites and some Lepidoptera, bacteria occur in the midgut which, in these insects is strongly acidic (Bauer et al. 2000; Shannon et al. 2001). The occurrence of Proteobacteria, isolated mainly from the midgut in fruit flies has been reported in several publications (Drew and Lloyd 1991; Kuzina et al. 2001; Marchini et al. 2002; Murphy et al. 1994), and this finding contrasts with the bacterial communities of other insects, e.g. termites, which have three major groups of microorganisms, Bacteroidetes, Firmicutes and Spirochaetes (Miyata et al. 2007).

The results in this study indicated that the crop and midgut were inhabited by different groups of bacteria. However, both *B. cacuminata* and *B. tryoni* possessed similar bacteria genera and species. The Firmicutes were found in the alimentary tract of adult wild *Bactrocera* species for the first time, demonstrating that the gut bacteria of fruit flies do not belong primarily to the Proteobacteria, especially Enterobacteriaceae as previously thought. Indeed, the rarefaction analyses confirmed a very high level of microbial diversity, especially with regard to bacteria, in the fruit fly species studied. This study has been the first to use 16s rRNA gene sequencing on tropical tephritid species and the bacterial community in the adult fly gut proved to be more diverse than that reported in species of Coleoptera (Delalibera et al. 2007; Vasanthakumar et al. 2006, 2008) and our rarefaction analyses indicated a bottomless pit of microbial diversity.

The phylogenetic analysis, demonstrated through the phylogenetic tree, that most of the fruit fly bacteria isolated are taxonomically close to known species listed on databases. In particular, isolates within class Gammaproteobacteria, family Enterobacteriaceae, were similar to known species. In the other four classes of bacteria, some of the isolated bacteria appear to be similar to but different from known species. Further research is required on both the taxonomic identity of these fruit fly associated bacteria as well as the biological relationships between fruit flies and bacteria, especially the lactic acid bacteria in the class Firmicutes.
